# Editorial: New e-health interventions and diabetes: effects on self-management, psychological well-being and quality of life

**DOI:** 10.3389/fcdhc.2023.1340396

**Published:** 2023-11-29

**Authors:** María Teresa Anarte-Ortiz

**Affiliations:** Department of Personality, Assessment and Psychological Treatment, Instituto de Investigación Biomedica de Málaga (IBIMA), University of Malaga, Malaga, Spain

**Keywords:** e-health, diabetes, intervention, self-management, well - being, quality of life

Diabetes is a chronic disease that has a great impact on people who live with it, as it requires a delicate balance between diet, exercise, self-monitoring of glucose and the administration of insulin to achieve adequate glycemic control. This involves continuous decision-making (especially in type 1 diabetes), which affects lifestyle and can generate psychological discomfort with a significant impact on quality of life.

Many factors intervene in good self-management of diabetes. However, of all of these, a good psychological state, as well as appropriate strategies for self-care and decision-making appear to be fundamental. Diabetes healthcare management should include specific strategies for the early detection and treatment of mental health problems to reduce their impact on health outcomes. In this sense, new technologies offer great potential.

Information and communication technologies (ICT) have led to a social revolution that is affecting all levels of people’s lives, even health. The World Health Organization defines eHealth as the use of ICT for health. Thus, it uses electronic means to support the delivery of health services and the management of health systems that provide health benefits and the cost to achieve them (1).

Advances in technologies associated with diabetes care have changed diabetes treatment, providing a great opportunity to patients with type 1 diabetes by facilitating daily diabetes self-care and, therefore, improving quality of life. The new e-health interventions include both new information and communication technologies (ICTs) applied to health and e-health interventions (such as new insulin pumps, CGM’s or closed loop systems).

Most of the digital solutions developed have focused on the monitoring of physical factors, diet, physical activity, and adherence to prescribed medication (2, 3). However, the impact of psychosocial factors should also be studied. Many types of eHealth interventions have demonstrated improvements in self-management behaviors, psychosocial outcomes (depression, anxiety, and distress), psychological well-being and clinical measures such as HbA1c (4–7).

New e-health interventions have been rapidly growing and evolving in the field of diabetes. This growth has been so rapid that further scientific evidence is necessary. In this regard, the systematic review by Morris et al. (8) reports no clear evidence of an impact of digital health interventions on health and social care utilization or costs; therefore additional research is needed.

In addition, the proliferation of digital health solutions requires standardized frameworks for implementing diabetes data and technology solutions. Although frameworks exist, they focus primarily on clinical implementation, and less on the process of integrating the new technology into the clinical setting.

The quality of life evidence for technology is positive overall, but for some people the burdens of technology outweigh the benefits and create barriers to uptake (9, p. 23).

Accordingly, further scientific evidence is needed regarding these advances in the functionality and application of new e-health interventions in diabetes.

This Research Topic includes both studies that provide novel empirical evidence for new e-health interventions (one in young people and two in adults) and a framework for integrating diabetes data and technology solutions that can guide clinicians implementing this new technology.

The first article evaluates the well-being and satisfaction of youth and their parents with diabetes care using mobile blood glucose self-monitoring technology and family-centered goal setting.

Continuous glucose monitors allow us to track glucose variability in the real-world environment but are stressful for the patient. In a second study of this Research Topic, an intervention based on the stress management and resiliency training (SMART) program is analyzed.

Another study analyzes whether Internet-based cognitive behavioral therapy to reduce depressive symptoms in type 1 diabetes is effective.

Finally, an interesting study proposes a practical and simplified framework to implement integrated diabetes data and technological solutions based on five interconnected phases.

Although these quality studies respond to the objectives of this Research Topic and represent an advance in the knowledge of this Research Topic, further progress is still possible with new studies, since eHealth is a promising complement to diabetes care that poses important challenges that must be studied.

## Author contributions

MA: Writing – original draft, Writing – review & editing.
